# A Closer Look at EGFR Inhibitor Resistance in Non-Small Cell Lung Cancer through the Lens of Precision Medicine

**DOI:** 10.3390/jcm12051936

**Published:** 2023-03-01

**Authors:** Martin Sattler, Isa Mambetsariev, Jeremy Fricke, Tingting Tan, Sariah Liu, Nagarajan Vaidehi, Evan Pisick, Tamara Mirzapoiazova, Adam G. Rock, Amartej Merla, Sunil Sharma, Ravi Salgia

**Affiliations:** 1 Department of Medical Oncology, Dana-Farber Cancer Institute, 450 Brookline Ave., Boston, MA 02215, USA; 2 Department of Medicine, Harvard Medical School, Boston, MA 02115, USA; 3 Department of Medical Oncology and Therapeutics Research, City of Hope, 1500 E Duarte Road, Duarte, CA 91010, USA; 4 Department of Computational and Quantitative Medicine, City of Hope, 1500 E Duarte Road, Duarte, CA 91010, USA; 5 City of Hope Chicago, 2520 Elisha Avenue, Zion, IL 60099, USA; 6 Division of Applied Cancer Research and Drug Discovery, Translational Genomic Research Institute (Tgen), 445 N 5th St, Phoenix, AZ 85004, USA

**Keywords:** EGFR, non-small cell lung cancer, drug resistance, genetic/nongenetic, epigenetics

## Abstract

The development of EGFR small-molecule inhibitors has provided significant benefit for the affected patient population. Unfortunately, current inhibitors are no curative therapy, and their development has been driven by on-target mutations that interfere with binding and thus inhibitory activity. Genomic studies have revealed that, in addition to these on-target mutations, there are also multiple off-target mechanisms of EGFR inhibitor resistance and novel therapeutics that can overcome these challenges are sought. Resistance to competitive 1st-generation and covalent 2nd- and 3rd-generation EGFR inhibitors is overall more complex than initially thought, and novel 4th-generation allosteric inhibitors are expected to suffer from a similar fate. Additional nongenetic mechanisms of resistance are significant and can include up to 50% of the escape pathways. These potential targets have gained recent interest and are usually not part of cancer panels that look for alterations in resistant patient specimen. We discuss the duality between genetic and nongenetic EGFR inhibitor drug resistance and summarize current team medicine approaches, wherein clinical developments, hand in hand with drug development research, drive potential opportunities for combination therapy.

## 1. EGFR Mutations in Cancer

**Figure 1:**
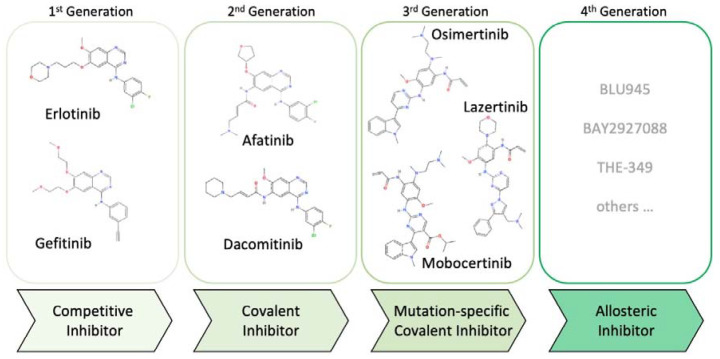
Evolution of EGFR tyrosine kinase inhibitors.

The *epidermal growth factor receptor* (*EGFR*) gene encodes for a transmembrane tyrosine kinase, is expressed in many tissues at various levels and is normally activated by its ligand epidermal growth factor [1]. Alterations of EGFR are common in solid tumors, including amplifications and activating mutations, in particular for patients with glioblastoma and non-small cell lung cancer (NSCLC). There are striking differences in NSCLC patients, with a significantly higher frequency of EGFR mutations in patients of East Asian heritage versus Caucasian patients and somewhat higher incidence in women and never-smokers [2,3,4]. About 90% of the identified activating EGFR mutations in lung adenocarcinoma involve either the L858R substitution in exon 21 or in-frame deletions in exon 19, leading to malignant transformation with ligand-independent activation of growth and anti-apoptotic pathways. Additional rare EGFR alterations include point mutations, insertions or deletions in exon 18–21 [5,6]. The development of ATP-competitive small-molecule EGFR inhibitors demonstrated the significance of these EGFR mutations for cancer growth. Gefitinib was the first molecularly targeted EGFR inhibitor that showed remarkable efficacy in lung cancer patients with EGFR mutations [7,8] (Figure 1). Currently, different FDA-approved EGFR inhibitors are available, and it appears that common and uncommon oncogenic EGFR mutations are preferably inhibited by some of these drugs [5]. Unfortunately, as it is the case for most small-molecule inhibitors that target oncogenic tyrosine kinases, resistance eventually ensues. An initial major focus was identifying on-target mutations in the drug binding pocket of the inhibitor. Depending on the mechanisms of action of the drug, mutations can also occur in other parts of EGFR and it appears that resistant mutations are context-specific. It is also possible that transformation in resistant cells is driven by off-target genetic alterations that lead to the activation of other transforming proteins and circumvent EGFR dependency. However, a significant portion of EGFR inhibitor resistance is driven by mechanisms that have not yet been identified and it has become clear that nongenetic mechanisms of resistance may play a larger role than previously thought. In this review, we take a closer look at the various genetic and nongenetic mechanisms of resistance towards EGFR inhibitors, both of which can either be acquired or pre-existing. The overall mechanism of how clonal selection occurs may be similar between the drug resistance mechanism towards different classes of drugs and towards different drug targets in growth pathways. We have previously suggested an intermediate drug-tolerant state for acquired genetic as well as nongenetic KRAS inhibitor resistance that may also apply here for the occurrence of genetic and nongenetic mechanisms of EGFR inhibitor resistance [9,10]. Nevertheless, the molecular mechanisms that determine the resistance pathways are not known. It has become apparent that EGFR-targeted monotherapy, even with next-generation inhibitors, is prone to drug resistance. Initially, research was focused on catching up to emerging resistance mutations and now it appears that combination therapy may be a more viable approach to at least delay treatment failure and increase clinical benefit. Within this context, we will further summarize recent team medicine approaches directed towards overcoming EGFR inhibitor resistance.

## 2. Evolution of EGFR Inhibitors

First-generation small-molecule EGFR inhibitors (erlotinib, gefitinib) were designed to interfere with EGFR tyrosine kinase activity by competing with binding of the adenine base of ATP to its binding pocket. The core interacting residues for ATP in EGFR include L718, V726, A743, M793 and L844 [11] and many competitive inhibitors commonly form interactions, in particular with M793 at the hinge region, including gefitinib and erlotinib [12]. About half the patients treated with these drugs acquire the T790M mutation at the highly conserved ‘gatekeeper’ residue and it has been suggested that future covalent inhibitors may circumvent this escape mechanism [13,14,15]. Indeed, second-generation inhibitors (afatinib, dacomitinib) were designed to be structurally related to these compounds but contained additional moieties to facilitate covalent binding to C797, in addition to the inhibition of the EGFR tyrosine kinase activity. Unfortunately, these drugs are also susceptible to the emergence of T790M mutations as they cannot distinguish between wild-type and mutant EGFR. This broad inhibition of EGFR is associated with dose-limiting side-effects, which do not allow for sufficient inhibition of the T790M mutation [16,17]. Mutant-selective third-generation inhibitors (osimertinib, lazertinib) were a true product of team medicine, where efforts from clinicians, clinical scientist, medicinal chemists, pathologists, biostatisticians and others led to the design of new EGFR inhibitors to circumvent these limitations and to specifically target EGFR. These contain activating mutations, including the T790M resistance mutation [18,19]. Even though these inhibitors do not target wild-type EGFR and have therefore little side-effects associated with this particular target, they are also susceptible to on-target drug resistance, such as C797S mutations or others.

In 2018, osimertinib became the first drug in this class to receive FDA-approval for first-line treatment of metastatic NSCLC with EGFR exon 19 deletions or exon 21 L858R mutations. The double-blind, phase III FLAURA trial with 556 patients established the efficacy of osimertinib, demonstrating prolonged progression-free survival [20]. When compared to standard EGFR inhibitor therapy, osimertinib increased median progression-free survival from 10.2 months to 18.9 months (hazard ratio (HR) 0.46; 95% CI: 0.37 to 0.57). Both, standard EGFR inhibitor therapy and osimertinib had similar objective response rates (76% vs. 80%), but the median duration of response with standard EGFR inhibitor therapy was 8.5 months (95% CI, 7.3 to 9.8) and osimertinib resulted in a 17.2-month response (95% CI, 13.8 to 22.0). Additionally, standard EGFR inhibitor therapy had a higher rate of grade 3/4 adverse reactions compared to osimertinib (45% vs. 34%). A further long-term follow up also demonstrated increased overall survival with osimertinib, compared to standard EGFR inhibitor therapy, in previously untreated patients [21]. These results further suggest that adverse reactions with osimertinib maybe somewhat higher (42% vs. 47% in the standard EGFR inhibitor therapy group) than previously reported. In general, osimertinib increased overall survival by almost 7 months to 38.6 months (95% CI: 34.5 to 41.8) compared to the standard therapy group result at 31.8 months (95% CI: 26.6 to 36.0). After 3 years, 28% (79/279; 20.7 months median exposure) of osimertinib treated patients were still on trial, versus 9% (26/277; 11.5 months median exposure) in the comparison group. A meta-analysis of 15 studies with 324 patients further supported a role for osimertinib in the control of intracranial metastatic disease, with complete intracranial response rates of 7% to 23% [22]. The objective response rate was calculated for 195 patients at 64% (95% CI: 53–76) and the disease control rate was 90% (95% CI: 85–93), calculated for 246 patients.

The efficacy of osimertinib in EGFR-mutated NSCLC patients harboring exon 20 insertion mutations (up to 12% of mutated EGFR) is limited and this population can benefit from mobocertinib, a C797 covalent EGFR inhibitor, which may be resistant to C797S mutations as well [23,24,25]. In 2021, mobocertinib was FDA-approved as the first drug for NSCLC patients with locally advanced or metastatic disease that had an EGFR exon 20 insertion mutations and progressed with platinum-based chemotherapy. The rate of adverse events was found to be similar to that of other EGFR inhibitors and in general manageable. In the phase I/II dose-escalation/expansion trial a response rate was achieved in 43% (12/28) of the patients (95% CI: 24 to 63) and the median progression-free survival was 7.3 months [24]. Further, in a larger cohort of 114 patients, the objective response rate was 28% (95% CI: 20 to 37), the median progression-free survival was 7.3 months (95% CI: 5.5 to 9.2) and median overall survival was 24.0 months (95% CI: 14.6 to 28.8) [26]. Additional data suggest that the intracranial activity of mobocertinib could be limited. Mobocertinib may provide better responses in patients without brain metastases, who benefited from longer treatment periods, and the intracranial anti-tumor activity appears to be insufficient [27].

Lazertinib was tested in a phase I/II clinical trial with 38 patients in the dose escalation group and 89 patients in the dose expansion group, where it was generally well tolerated. Treatment-related grade 3 or 4 adverse events occurred in only 3% (4/127) of patients, without any events that lead to death or treatment-related deaths. A total of 54% (69/127) of patients achieved an objective response (95% CI: 46 to 63) [19]. In another South Korean phase 1/2 study with 78 T790M-positive NSCLC patients, lazertinib caused a complete response in one patient and 53.9% (41/78) of the patients had partial responses, resulting in a similar objective response rate of 55.3% (95% CI: 44 to 66) [28]. Median progression-free survival was 11.1 months (95% CI: 5.5 to 16.4) and the median overall survival did not reach 22 months. As expected, loss of EGFR T790M was identified as a major resistance mechanism. Lazertinib was also active in the brain and suppressed intracranial tumor growth, with one patient showing a complete response and five patients showing partial responses, resulting in an intracranial objective response rate of 85.7% (95% CI: 60 to 100.0). Lazertinib received local approval in South Korea in 2021 for NSCLC patients with EGFR T790M mutations that had previously received treatment with EGFR inhibitors and that had locally advanced or metastatic disease. However, it has not yet gained FDA approval in the USA.

Fourth-generation allosteric mutant-selective EGFR inhibitors that have different binding sites are currently being tested. These mutations can be compounded and include the activating mutation (e.g., exon 19 deletion, L858R), the first-/second-generation inhibitor resistance mutation (e.g., T790M) and/or an osimertinib resistance mutations. Occasionally, mutations that cause osimertinib resistance may not necessarily emerge from clones that contain the T790M resistance mutations but can also originate from the original clone containing the oncogenic mutation, such as L858R with the M766Q exon 20 resistance mutation. Interestingly, this double-mutant can be sensitive towards the tyrosine kinase inhibitor neratinib, which was originally developed against EGFR family members [29]. The occurrence of multiple mutations in EGFR complicates the development of next generation inhibitors but allosteric mutant-selective fourth generation EGFR inhibitors are designed to show efficacy in this context. Whether they must be combined with other targeted therapies, standard chemotherapy or immunotherapy will have to be determined. Team medicine takes center stage in the development of new therapeutics that are essentially initiated by results from precision medicine approaches. The development of second-generation EGFR inhibitors has demonstrated that pre-clinical results may not easily be transferrable to clinical practice and that with a combined effort of various pre-clinical and clinical groups of scientist significant progress can be achieved.

## 3. Genetic Mechanisms of EGFR Inhibitor Resistance beyond On-Target Mutations

**Figure 2:**
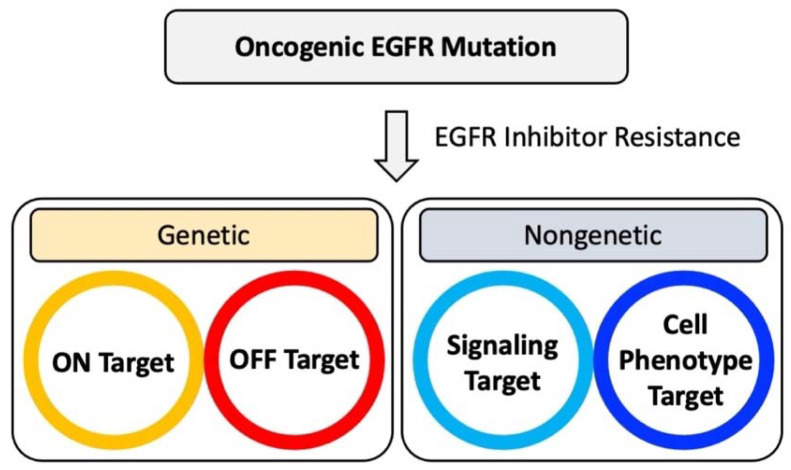
Major genetic and nongenetic escape mechanisms for acquired EGFR inhibitor resistance.

Different on-target mutations within EGFR have been reviewed previously (e.g., [30]). Additional genetic changes frequently target the signaling molecules that substitute for the functional activation of pathways, which are otherwise dysregulated by oncogenic EGFR (Figure 2). Mutated proteins within these pathways can present themselves as therapeutic targets or hint at potential targets for combination therapy.

**Figure 3:**
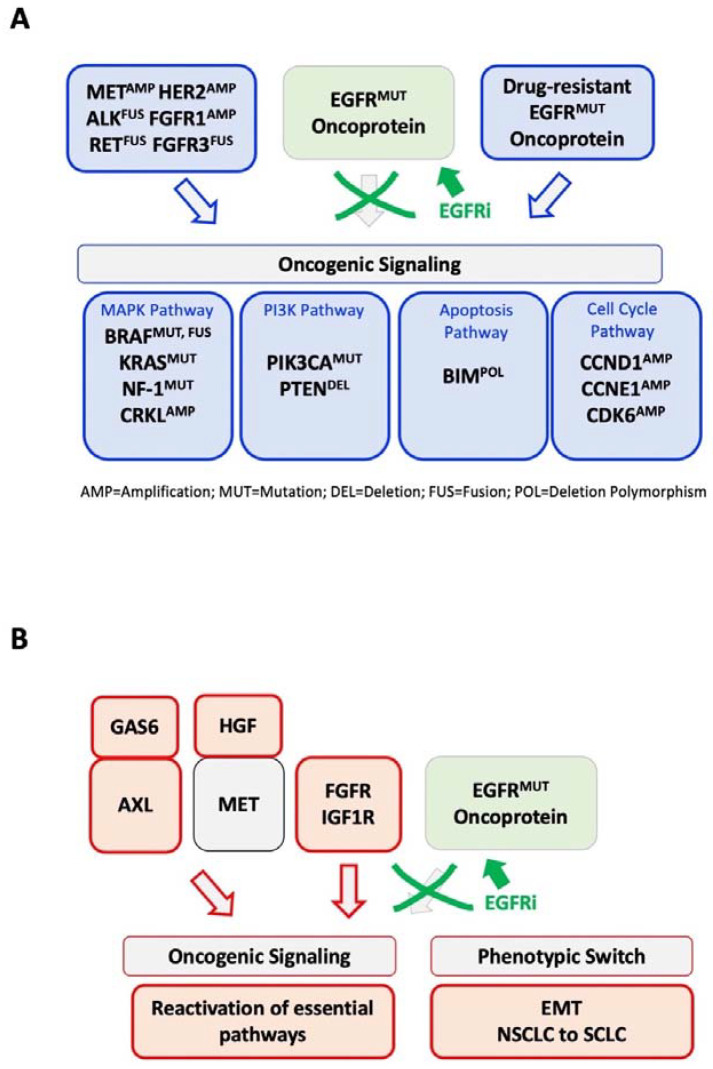
Model of genetic and nongenetic drug resistance mechanisms. Simplified model of possible (**A**) genetic and (**B**) nongenetic alteration identified in patients with therapy-related resistance to EGFR inhibitors.

There are at least two categories of signaling targets: (a) genetic alterations of receptors that substitute for EGFR signaling and (b) mutations within signaling pathways activating mechanisms downstream of EGFR (Figure 3). The first category includes amplification of the gene for the receptor tyrosine kinases (RTKs) MET, HER2 and FGFR1 or the activating fusion of ALK, FGFR3 and RET, essentially substituting for loss of EGFR kinase activity [31,32,33,34,35,36,37]. The second mechanism affects downstream effectors, including targets within the mitogen-activated protein kinase (MAPK) pathway (*BRAF* mutation and fusion [36,38], *KRAS* mutation [39,40]), *CRKL* amplification [41]), the phosphatidylinositol-3-kinase (PI3K) pathway (*PIK3CA* (phosphatidylinositol-3-kinase catalytic α subunit) mutation [42], *PTEN* deletion [43]), or cell cycle pathways (CCNE1, CCND1, CDK6 [44]) were observed. There is also a reduced expression of the tumor suppressor NF-1, a KRAS-specific GTPase-activating protein (GAP), but whether these changes are genetic and/or nongenetic has not been well established. However, concomitant exon 19 deletion and stop-gain mutation in NF1 can lead to poor clinical activity of gefitinib and osimertinib, suggesting that these mechanisms may be involved in EGFR inhibitor resistance [45,46]. Further, BIM deletion polymorphisms did not directly cause EGFR inhibitor resistance but resulted in significantly shorter progression-free survival and therefore affected the overall efficacy of the treatment [47]. Additional rare mutations have been found in various models of cell line-based EGFR inhibitor resistance.

## 4. The EGFR Inhibitor Genetic Resistance Gap

Not all mechanisms of resistance are based on genetic changes that can be attributed to mutations (Figure 2). There is no defined overall proportion of specific mechanisms and it also likely depends on multiple factors, such as patient selection, pretreatment or cotreatment, and the type and class of EGFR inhibitor used, to name a few variables. For example, in a cohort of 37 patients resistant to first-generation EGFR inhibitors, 44% showed a nongenetic mechanisms of drug resistance (including phenotypic changes) [42]. First-line osimertinib resistance in NSCLC can be caused by 53–69% and second-line osimertinib resistance can be caused by 30–60% of unknown mechanisms that are likely mostly nongenetic [48]. Moreover, there is little information about what causes the regulation of signaling molecules through nongenetic mechanisms of EGFR inhibitor resistance. It is likely that these mechanisms involve typical modifications that regulate gene expression, such as changes in DNA methylation or changes in histone modifications, ultimately changing DNA accessibility and allowing for changes in gene expression. These alterations are unlikely to affect single genes but may contain unique vulnerabilities that could be exploited.

A complex and poorly understood mechanism of EGFR inhibitor resistance involves transformation into new histologic subtypes, including epithelial-to-mesenchymal transition (EMT) [42,49] and small cell lung cancer (SCLC) transformation of NSCLC cells [42,50] (Figure 3). The altered tumor cells are potentially substituting EGFR-dependency with other mechanisms that also lead to phenotypic changes. These mechanisms are expected to involve transcription factors and their effectors, which could be opportune targets for drug development in this patient population. Nevertheless, EGFR inhibitor-resistant cells, when transformed into SCLC, are genetically diverse and acquired resistant mutations may play a larger role in them than initially thought. The current standard-of-care SCLC therapy is utilized in this population, but overall survival is significantly lower than that of the non-transformed EGFR population and more therapeutic options specific to this population are required [42,51,52,53].

Similar to the genetic mechanism of resistance, nongenetic mechanisms also involve activation of other RTKs and their ligands, including increased expression of hepatocyte growth factor and the ligand for MET [54,55], as well as the upregulation of fibroblast growth factor receptors (FGFR) [56,57] and insulin-like growth factor 1 receptor (IGF1R) [58] in cell line models or AXL and its ligand GAS6 [49] in EGFR inhibitor-resistant cells. Another interesting mechanism involves the function of Aurora kinase A (AURKA) in the development of EGFR inhibitor resistance. Both AURKA and the related AURKB can share oncogenic features but possess different substrates, and there is considerable interest in targeting Aurora kinase activity in cancers [59]. AURKA is thought to induce at least some level of drug tolerance towards third-generation EGFR inhibitors, which can be reverted by AURKA inhibitors [60]. AURKB may play a more prominent role in EMT-transformed EGFR inhibitor resistance, where it is thought that its co-inhibition with EGFR enhances BIM- and PUMA-mediated apoptosis [61]. Even though not defined in EGF inhibitor resistance models, the activation of signal transduction and activator of transcription 3 (STAT3) [62], the RIG-I-TBK1-IRF3 axis [63] or nuclear factor-κB (NF-κB) [64] may induce residual signaling during the inhibition of EGFR in dependent cells that could be sufficient for the evolution of resistant clones.

## 5. Clinical Strategies in the Treatment of EGFR Inhibitor Resistance

Clinical strategies for third-generation EGFR inhibitor resistance are mainly focused on combination therapies that inhibit emerging off-targets or on trying to inhibit EGFR with on-target mutations at C797, the binding site of covalent EGFR inhibitors in NSCLC and glioblastoma (Table 1). Combinations include the inhibition of oncogenic EGFR with osimertinib or lazertinib and the targeting of MET with tepotinib (NCT03940703, NCT05120960) and savolitinib (NCT03944772) or the FDA-approved bispecific EGFR-MET antibody amivantamab, respectively (NCT05299125, NCT02609776). MET could also be targeted outside of clinical trials with FDA-approved drugs that are active against this RTK, including crizotinib or capmatinib. Other RTKs that are targeted in combination with osimertinib are mainly in line with resistance mechanisms that are described above and include ALK with alectinib (NCT03944772), RET with selpercatinib (NCT03944772) or HER2 with trastuzumab (NCT04285671) or EGFR, HER2, or HER4 with dacomitinib (NCT03755102). Additional approaches involve combinations with traditional chemotherapy (pemetrexed plus platinum chemotherapy) (NCT03944772, NCT05153408, NCT05299125, NCT02609776) or targeting cancer dependency pathways that are known to be activated downstream of EGFR, including cell cycle (CDK4/CDK6) (NCT04545710), MAPK pathway (NCT03944772, NCT03944772), PI3K pathway (NCT05284994) and others, depending on the resistance mechanism. The development of 4th-generation EGFR inhibitors or inhibitors that are active in the presence of C797 mutations are exciting, including BLU-945 (Blueprint Medicines), WJ13405 (Suzhou Junjing BioSciences), BAY2927088 (Bayer), JIN-A02 (J Ints Bio), HS-10375 (Jiangsu Hansoh Pharmaceutical), QLH11811 (Qilu Pharmaceutical), BPI-361175 (Xcovery Holding Company), and BDTX-1535 (Black Diamond Therapeutics). None of these drugs have yet been approved and little is known about their efficacy, but preclinical information published for BLU-945 [65] or BDTX-1535 [66] is promising and there are additional drugs that will reach clinical stage soon, such as THE-349 (Theseus Pharmaceuticals). It will be important to see whether genetic and nongenetic mechanisms of resistance will apply for these drugs as well and whether there is a significant increase in overall survival. Nongenetic mechanism are difficult to discern, and better biomarker strategies are needed to identify therapeutic targets.

Traditionally, immune checkpoint inhibitors did poorly as first-line therapeutic treatment in patients with oncogenic EGFR mutations. However, in certain contexts, some patients do benefit from reactivating the T-cell immune response [67]. Nevertheless, this class of therapeutics is also considered for the EGFR inhibitor resistance NSCLC population, including the anti-PD-1 antibody pembrolizumab (NCT03515837) and the anti PD-L1 antibody durvalumab (NCT03944772). Combinatory therapeutic options of EGFR inhibitors with immunotherapy in advanced NSCLC have been reported to result in an increase in the amount of grade 3 or higher toxicities, most notable pneumonitis, with no significant improvement in survival or response [68,69,70]. However, combination immunotherapy with chemotherapy plus antiangiogenics may be a more viable path towards the development of therapeutics. IMpower150 evaluated atezolizumab (anti PD-L1) with bevacizumab (anti VEGF-A) plus chemotherapy (carboplatin plus paclitaxel) in first-line nonsquamous NSCLC, and EGFR patients showed significantly improved progression-free survival and overall survival [71]. Even so, the majority of EGFR patients still receive TKI first-line therapy and evaluation of immunotherapy with chemotherapy plus antiangiogenic agents following the persistence of resistance is still ongoing. The ORIENT-31 evaluated sintilimab (anti PD-1) immunotherapy with bevacizumab plus chemotherapy (cisplatin plus pemetrexed) in EGFR patients following TKIs and results showed improved progression-free survival of 9.8 months and a response rate of 44% as compared to chemotherapy alone [72]. Overall, these results suggest immunotherapy, chemotherapy, and antiangiogenic therapy combinations may be the most promising therapeutic options. However, we must await more trial results to definitively determine the role of immunotherapy in the resistance setting.

## 6. Conclusions and Future Direction

The molecular mechanisms that cause genetic or nongenetic drug resistance are unknown, making it difficult to predict treatment strategies. There is some overlap between these two mechanisms, such as the activation of bypass RTK pathways, but there are also unique phenotypic changes induced by nongenetic mechanisms. There is currently no EGFR inhibitor that halts disease progression or even provides curative benefits due to drug resistance. A major goal of clinical strategies involves the identification of on-target and off-target mutations. These mutations allow for the maintenance of oncogenic signaling, either by blocking inhibitor binding or targeting bypass mechanisms. Liquid biopsies may help to provide useful insights into the type of mutations and will lead to the use of possible bypass pathway inhibitors. Additional tests that capture nongenetic changes should be considered for patients where no acquired driver mutations can be identified. Team medicine will lead the way and identify the best treatment strategies for novel or current therapeutics and evaluate their risk–benefit relationship, pinpoint novel escape mechanisms through precision medicine, evaluate and adjust treatments for differences in ethnicity, sex or age through clinical trials, provide the best possible care for patients while optimizing quality of life during treatment, and attempt to make affordable care available to all patients. Current common therapeutic approaches include combinations with drugs that target (a) bypass mechanism, such as MET inhibitors in cells with MET amplification, (b) common cancer pathways, such as apoptosis, cell cycle or MAPK pathways, (c) critical downstream effectors of EGFR, and (d) anti-folate and/or platinum chemotherapy. Alternatively, antibody drug conjugates (ADCs) and experimental vaccines can also be considered. The goal is not only to target cancer cells more efficaciously but possibly also to eliminate potential emerging drug resistant subclones to at least delay disease progression. A question that has been discussed for other oncogenes is that, in the presence of an inhibitor, the scaffold function of the mutated oncoprotein may contribute to some oncogenic signaling and therefore targeted degradation of EGFR should be considered [73]. Many inhibitors for bypass mechanisms are already available and there is hope that better biomarker strategies will help to identify the patient population that can benefit from these therapeutics, in particular for those with nongenetic resistance against EGFR inhibitors.

## Figures and Tables

**Table 1:** Ongoing or planned registered clinical trials of patients with resistance to 3rd generation EGFR inhibitors (query date: 3 January 2023). Indicated are oncogenic EGFR-targeted combination therapies (top) and their targets and matching therapeutic as well as monotherapies (bottom) targeting EGFR with mutations at C797 (C797X).

Primary Target	Primary Therapeutic	Secondary Target	Secondary Therapeutic	ClinicalTrials.gov Identifier
EGFR	Osimertinib	CDK4/CDK6	Abemaciclib	NCT04545710
EGFR	Osimertinib	mTOR Aurora A	Sapanisertib Alisertib	NCT04479306
EGFR	Osimertinib	Anti-EGFR	Necitumumab	NCT02496663
EGFR	Osimertinib	MET	Tepotinib	NCT03940703
EGFR	Osimertinib	MET	Tepotinib	NCT05120960
EGFR	Osimertinib	COX1/COX2 (AKT/BIM)	Aspirin	NCT04184921
EGFR	Osimertinib	MET	Savolitinib	NCT03944772
		EGFR	Gefitinib	
		Anti-EGFR	Necitumumab	
		Antifolate + Anti-PD1	Pemetrexed + Durvalumab	
		ALK	Alectinib	
		RET	Selpercatinib	
		Antifolate + Platinum Chemotherapy	Pemetrexed + Carboplatin or Cisplatin	
		MEK1/MEK2	Selumetinib	
		TROP2 ADC	Datopotamab-deruxtecan	
-	-	Topoisomerase + Anti PD-L1 + Platinum Chemotherapy	Etoposide + Durvalumab + Carboplatin or Cisplatin	
EGFR	Osimertinib	BCL-2/BCL-xL	Pelcitoclax	NCT04001777
EGFR	Osimertinib	BCL-2/BCL-xL	Navitoclax	NCT02520778
EGFR	Osimertinib	SRC	Dasatinib	NCT02954523
EGFR	Osimertinib	α/δ Phosphatidylinositol 3-kinase	TQ-B3525	NCT05284994
EGFR	Osimertinib	EGFR	Necitumumab +	NCT04285671
		HER2	Trastuzumab	
EGFR, HER2, HER4	Dacomitinib	EGFR	Alone or + Osimertinib	NCT03755102
EGFR-MET bispecific antibody	Amivantamab	EGFR	Lazertinib or	NCT05299125,
		Antifolate	+ Pemetrexed	NCT02609776,
		Chemotherapy	+ Carboplatin	NCT04077463
EGFR-MET bispecific antibody	EMB-01			NCT03797391
Anti-HER3 ADC	Patritumab Deruxtecan	EGFR	Osimertinib	NCT04676477
EGFR	Nazartinib (EGF816)	MEK1/MEK2	Trametinib	NCT03516214
PARP	Olaparib	Anti-PD-L1	Durvalumab	NCT04538378
Antifolate + Chemotherapy	Pemetrexed + Platinum Chemotherapy	Anti-PD-1	Alone or + Pembrolizumab	NCT03515837
EGFR (C797X)	BLU-701	EGFR	Alone or + Osimertinib	NCT05153408
		Antifolate	+ Pemetrexed	
		Chemotherapy	+ Carboplatin	
EGFR (C797X)	BLU-945	EGFR	Alone or + Osimertinib	NCT04862780
EGFR (C797X)	WJ13405			NCT05662670
EGFR (C797X)	BAY2927088			NCT05099172
EGFR (C797X)	JIN-A02			NCT05394831
EGFR (C797X)	HS-10375			NCT05435248
EGFR (C797X)	QLH11811			NCT05555212
EGFR (C797X)	BPI-361175			NCT05393466
EGFR (C797X)	BDTX-1535			NCT05256290

## Data Availability

Not applicable.
